# Bromodomain inhibitor i-BET858 triggers a unique transcriptional response coupled to enhanced DNA damage, cell cycle arrest and apoptosis in high-grade ovarian carcinoma cells

**DOI:** 10.1186/s13148-023-01477-x

**Published:** 2023-04-15

**Authors:** Marcos Quintela, David W. James, Agne Pociute, Lydia Powell, Kadie Edwards, Zoe Coombes, Jetzabel Garcia, Neil Garton, Nagindra Das, Kerryn Lutchman-Singh, Lavinia Margarit, Amy L. Beynon, Inmaculada Rioja, Rab K. Prinjha, Nicola R. Harker, Deyarina Gonzalez, R. Steven Conlan, Lewis W. Francis

**Affiliations:** 1 Swansea University Medical School, Swansea University, Singleton Park, Swansea, SA2 8PP UK; 2 Immunology Research Unit, GlaxoSmithKline, Medicines Research Centre, Stevenage, SG1 2NY UK; 3 Swansea Bay University Health Board, Swansea, SA12 7BR UK; 4 Cwm Taf Morgannwg University Health Board, Swansea, SA2 8QA UK; 5 Porvair Sciences Ltd, Wrexham, LL13 9XS UK

**Keywords:** Ovarian cancer, BETi, Advanced therapeutics, Drug development, i-BET858

## Abstract

**Background:**

Ovarian cancer has a specific unmet clinical need, with a persistently poor 5-year survival rate observed in women with advanced stage disease warranting continued efforts to develop new treatment options. The amplification of BRD4 in a significant subset of high-grade serous ovarian carcinomas (HGSC) has led to the development of BET inhibitors (BETi) as promising antitumour agents that have subsequently been evaluated in phase I/II clinical trials. Here, we describe the molecular effects and ex vivo preclinical activities of i-BET858, a bivalent pan-BET inhibitor with proven in vivo BRD inhibitory activity.

**Results:**

i-BET858 demonstrates enhanced cytotoxic activity compared with earlier generation BETis both in cell lines and primary cells derived from clinical samples of HGSC. At molecular level, i-BET858 triggered a bipartite transcriptional response, comprised of a ‘core’ network of genes commonly associated with BET inhibition in solid tumours, together with a unique i-BET858 gene signature. Mechanistically, i-BET858 elicited enhanced DNA damage, cell cycle arrest and apoptotic cell death compared to its predecessor i-BET151.

**Conclusions:**

Overall, our ex vivo and in vitro studies indicate that i-BET858 represents an optimal candidate to pursue further clinical validation for the treatment of HGSC.

**Supplementary Information:**

The online version contains supplementary material available at 10.1186/s13148-023-01477-x.

## Background

Ovarian cancer (OC) remains one of the five leading causes of cancer-related mortality in women worldwide, accounting for approximately 4000 deaths in 2017 in the UK [1, 2]. Elevated mortality rates can be attributed to the asymptomatic nature of early disease states as well as a lack of long-term effective treatment strategies for advanced conditions.

The term OC encompasses a variety of tumours with differing cells of origin that involve the ovary, of which epithelial represents the vast majority [3]. Epithelial carcinomas can be further subdivided into various histological subtypes that exhibit distinct clinical features, responses to chemotherapies and outcomes: high-grade/low-grade serous, endometrioid, clear cell and mucinous [4]. High-grade serous carcinomas (HGSC) are the most common, as they account for ~ 75% of all epithelial ovarian cancers, and are one of the most aggressive subtypes [5]. The genomic landscape of HGSC is characterised by universal mutations in the *TP53* tumour suppressor [6], alterations in a variety of genes involved in homologous recombination DNA repair pathways such as BRCA1/2 and widespread copy number alterations [7]. The surprisingly low abundance of recurrent somatic alterations highlights a need for individualised therapies. At present, only a few molecular targeted strategies such as PARP inhibitors aimed at patients with BRCA1/2 mutations (~ 20–25% HGSC) are yielding significant clinical benefits [8].

Altered patterns of epigenetic modifications, such as methylation and acetylation of histones, are common in many human cancers [9]. Mediators of histone acetylation including histone acetyl-transferases (HATs), histone deacetylases (HDACs) and readers of acetylation such as bromodomain (BRD)-containing proteins are often deregulated and constitute promising classes of therapeutic targets [10].

The most studied group of BRD-containing proteins are the bromodomain and extra-terminal domain (BET) family, comprised of BRD2, BRD3, BRD4 and BRDT, characterised by the presence of two tandem bromodomains (BD1/BD2) with preferential interaction towards diacetylated peptides present in histone tails [11, 12]. BET proteins are ubiquitously expressed across human cell types and have overlapping functions although they are not fully functionally redundant [13]. BRD2 and more so BRD4 (80% identity at the amino acid level) are known to have crucial roles involving cell cycle regulation, proliferation and DNA damage repair [14, 15], whereas little is known about the biological functions and the potential roles in disease of BRD3 [16]. BRD4 is considered to have a broader role in transcriptional activation and is regarded as the primary BET protein required to maintain steady state gene expression [13, 17]. However, all BET proteins are required for specific gene transcription and cooperation between them is required to efficiently induce gene expression following stimuli [18].

Several lines of evidence from preclinical studies indicate a role of BET proteins in cancer, providing the rationale for targeting BET proteins for the development of new anti-cancer drugs. Bromodomain inhibitors (BETi) are small molecule inhibitors aimed at blocking the interaction between BET proteins and acetylated lysines, resulting in displacement of bromodomain-containing proteins from chromatin. Several BETis have been developed by GSK and others, including JQ1 [19], i-BET726 [20] and i-BET151 [21]. Ongoing phase I and II clinical trials are evaluating the safety and efficacy of such compounds in a range of haematologic malignancies and solid tumours [22]. Importantly, BETi have been shown to induce significant clinical remissions whilst selectively inhibiting the transcription of only a few hundred genes in certain disease settings demonstrating their specificity [23, 24, 25].

The rationale for the use of BETi in OC is multi-faceted, and includes the presence of relatively frequent, recurrent focal gene amplification of BET-related oncogenes such as c-MYC, and more importantly of BRD4 itself [26]. BRD4 amplification occurs most frequently in HGSC patients (~ 18%) and is correlated with poor clinical outcomes [27]. The effects of BETi in OC seem to mirror events observed previously in different cancer backgrounds, eliciting significant anti-proliferative effects regardless of histological subtype [28, 29]. BETi-associated attenuation of OC cell growth has been linked mainly to cell cycle arrest and cellular apoptosis [28, 30, 31], although other affected cell functions like metabolism and oxidative stress have also been specified [28]. A significant number of signalling pathways known to be de-regulated in OC have been identified as potential targets of BETi, including FOXM1, JAK/STAT and the SWI/SNF remodelling complex [29, 32, 33].

On-going phase I/II clinical trials for several solid tumours including OC have shown favourable pharmacokinetic profiles with relatively high levels of toxicity [22], suggesting that increased efforts are needed in order to identify BETi that offer reduced toxicity and increased tumour specificity. i-BET858 is a bivalent pan-BETi with proven in vivo inhibitory activity and high selectivity towards the BET family over other bromodomains such as BRD7 and BRD9 (> 1,000-fold) that was originally linked to the development of autism-like syndrome in mice [34]. Because of the relatively limited efficacy of early BETi in OC models, we set out to elucidate whether i-BET858 displayed any enhanced levels of efficacy in HGSC. Viability assays performed in both ovarian cancer cell lines of HGSC origin as well as patient derived clinical samples revealed an enhanced cytotoxic activity of i-BET858 compared with other BETi. Whilst the mechanism of action of i-BET858 on DNA damage, cell cycle blockade and apoptosis did not differ significantly from that of other BETi, these mechanistic effects were induced at lower concentrations.

## Results

### i-BET858 inhibits cell viability in cellular models and primary patient derived samples

**Fig. 1:**
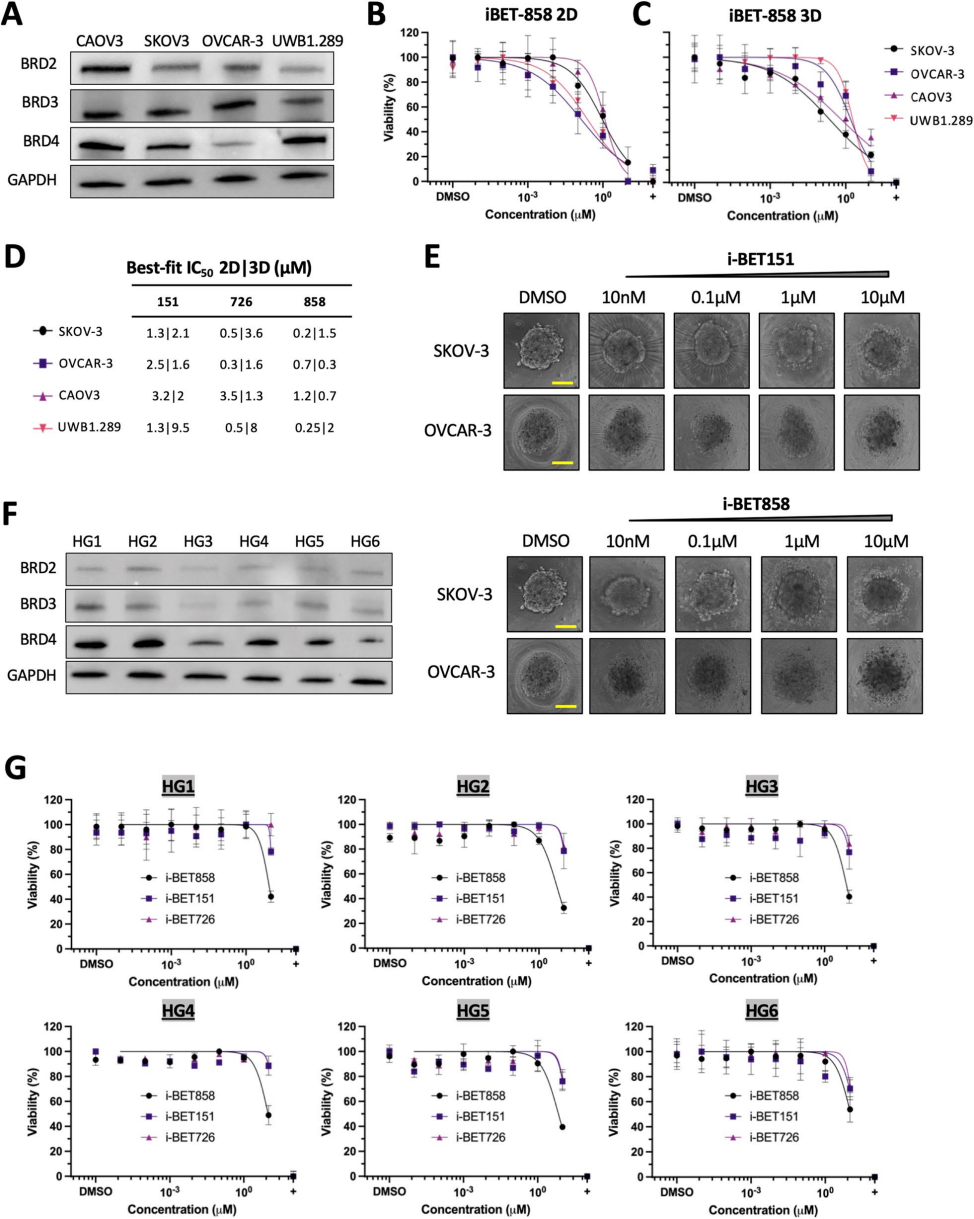
i-BET858 exhibits increased efficacy compared to other BETi in cell lines and primary samples. **A** Protein lysates from ovarian cancer cell lines were subjected to western blot analyses to study basal BET protein expression (BRD2, BRD3, BRD4). GAPDH was used as loading control. **B**, **C** and **D** Determination of IC_50_ values (µM) using 2D (**B**) and 3D (**C**) models of ovarian cancer cell lines. Cells were treated for 48 h with varying concentrations of i-BET858, i-BET151 and i-BET726 (10 pM–10 µM). DMSO was used as vehicle control, and staurosporine was used as positive control (+). (**E**) Microscopy images of 3D spheroids of SKOV3 and OVCAR-3 cells treated with varying concentrations of i-BET151 and i-BET858 (10 nM–10 µM) and vehicle control. Scales represent 100 µm. (**F**) Protein lysates from patient derived primary cells were subjected to western blot analyses to study basal protein expression of BRD2, BRD3 and BRD4. GAPDH was used as loading control. (**G**) Determination of IC_50_ values (µM) using 2D models of HGSC primary cells treated for 48 h with varying concentrations of i-BET858, i-BET151 and i-BET726 (10 pM–10 µM)

The effect of i-BET858 on cell viability was compared to the BETi compounds i-BET151 and i-BET726 using a panel of OC cell lines representative of HGSC (SKOV3, OVCAR-3, CAOV3, UWB1.289) that were cultured as monolayers (2D) and spheroids (3D). Initially, BRD2, BRD3 and BRD4 expression levels were determined in the cell line panel (Fig. 1A). All OC cell lines exhibited consistent expression levels of at least two BRDs, suggesting that any differences observed in half-maximal inhibitory concentrations (IC_50_) would not stem from differing target expression (Fig. 1B). Within 48 h of treatment, all three compounds significantly inhibited the growth of all OC cell lines in a dose-dependent manner (Fig. 1B and D). Compounds i-BET858 (IC_50_: 200 nM-1.2 µM) and i-BET726 (IC_50_: 300 nM-3.5 µM) displayed nanomolar efficiency on most monolayer cultures, whereas i-BET151 consistently exhibited micromolar efficiency (IC_50_: 1.3 µM-3.2 µM) (Fig. 1D). Overall, the efficacy of i-BET726 (IC_50_: 1.3 µM-8 µM) and i-BET151 (IC_50_: 1.3 µM-9.5 µM) on 3D spheroids was significantly lower (Fig. 1C and D). In contrast, i-BET858 exhibited nanomolar efficiency in 3D spheroids (IC_50_: OVCAR-3 300 nM; CAOV3 700 nM) (Fig. 1C–E), suggesting that higher potency, increased penetration, or both were achieved compared to other BETi compounds.

To evaluate whether comparative efficacy of i-BET858, i-BET151 and i-BET726 was similar in primary cells, an initial evaluation using six tumour derived samples from patients with diagnosed late stage HGSC (HG 1–6) was undertaken (Additional file 1: Table S2). Prior to BETi treatment, we assessed the expression levels of BRD2, BRD3 and BRD4 (Fig. 1F). As opposed to the panel of cell lines, patient derived cells primarily expressed BRD4 and exhibited low levels of BRD2 and BRD3. Primary cells were grown as monolayers and subsequently treated for 48 h with increasing doses of BETi. Similar results were observed for i-BET151 and i-BET726 (IC_50_ > 10 µM), whereas i-BET858 displayed a marginally enhanced cytotoxic activity in 5 of the 6 patient derived samples (HG1 = 8.5 µM, HG2 = 5.2 µM, HG3 = 6.7 µM, HG4 = 9.6 µM, HG5 = 6.9 µM, HG6 = 10 µM) (Fig. 1G). Whilst elevated levels of BET proteins including BRD4 have been previously linked with higher BET sensitivity in OC cells [33], the effects seen for i-BET858 did not appear to be linked to BRD levels, as cell viability did not correlate with BET expression across the clinical samples (Fig. 1G).

### Transcriptome profiling reveals distinct regulatory effects for i-BET858

**Fig. 2:**
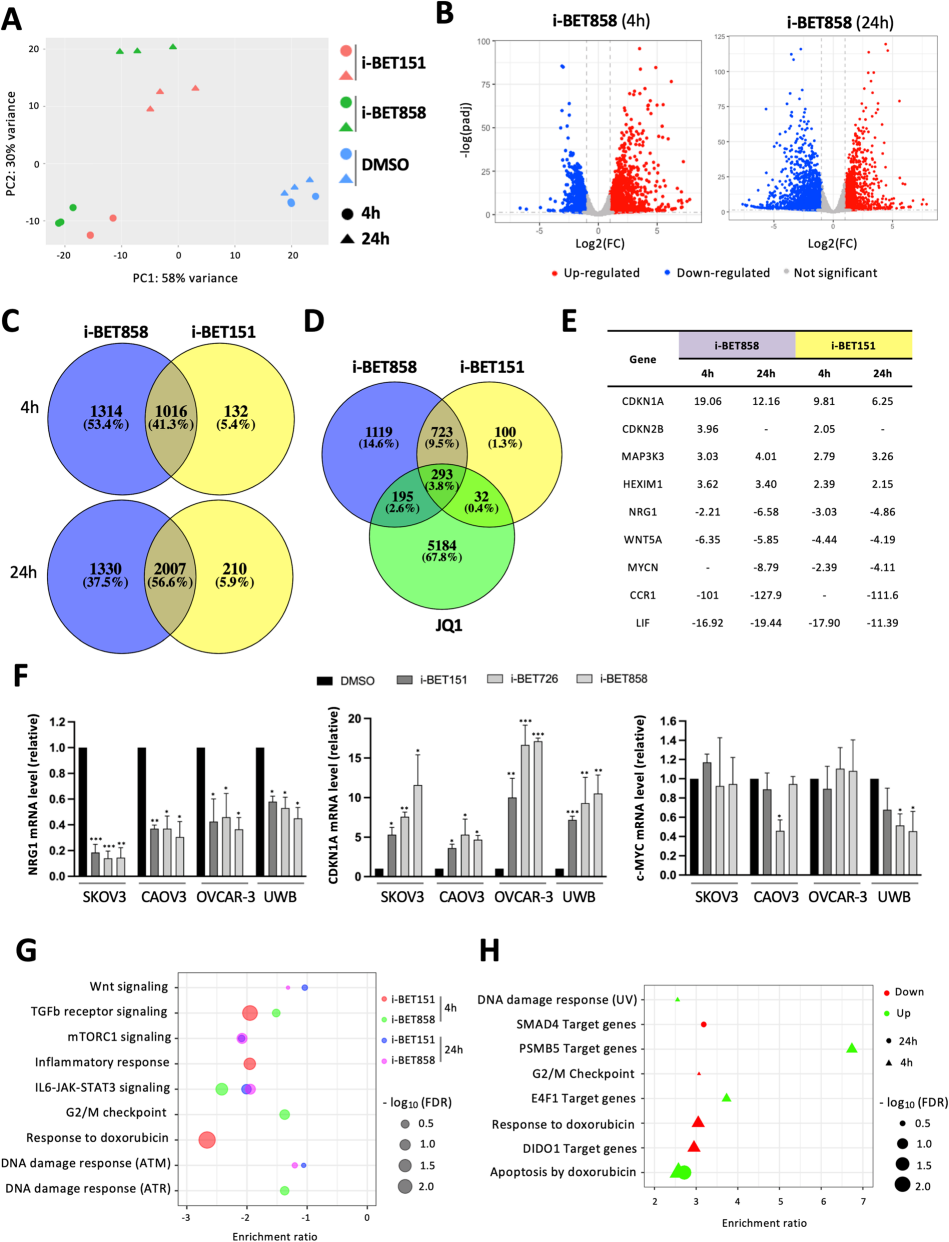
Transcriptome analyses of i-BET858 treatment highlight known BETi-associated pathways as well as unique features. **A** Principal component analysis (PCA) showing the distribution of data following RNA-sequencing of OVCAR-3 samples treated with i-BET858, i-BET151 and DMSO control for 4 and 24 h. Three biological replicates of each sample were sequenced. **B** Volcano plots displaying gene expression levels after 4 and 24 h of i-BET858 treatment (1 µM) in comparison with the DMSO control. Grey dots represent transcripts whose expression did not change significantly as a result of treatment, whilst red and blue dots represent transcripts that were up and down-regulated, respectively. **C** Venn diagram comparisons of differentially expressed genes between i-BET858 and i-BET151 treatments after 4 and 24 h. **D** Venn diagram comparison of differentially expressed genes between i-BET858 (4 h, 1 µM), i-BET151 (4 h, 1 µM), and JQ1 (0.125 µM, 40 min, GSE77568). **E** Table summarising expression changes on genes associated with a core BETi response. **F** Cell lysates of SKOV3, CAOV3, OVCAR-3 and UWB1.289 (UWB) cell lines were subjected to qRT-PCR validation to confirm changes in expression levels of NRG1, p21 and c-MYC targets. **G** Gene set enrichment pathway analysis of differentially expressed genes after 4 and 24 h of i-BET858 and i-BET151 treatments. **H** Gene over-representation pathway analysis of differentially expressed genes uniquely affected with i-BET858 treatment. All values represent the mean ± standard deviation (SD) of three biological samples (*P < 0.05, **P < 0.01, ***P < 0.001)

RNA-sequencing (RNA-seq) was undertaken on OVCAR-3 samples treated for 4 and 24 h with 1 µM of i-BET858 and i-BET151 to understand the comparative transcriptomic effects of i-BET858. Principal component analysis (PCA) revealed a significant separation between the controls (DMSO) and treatment samples (Fig. 2A), suggesting a genome-wide effect of both i-BET858 and i-BET151 on the OVCAR-3 transcriptome. Both BETi samples grouped on treatment duration (4-24 h) suggesting different time-dependent-related gene expression (Fig. 2A), consistent with a previous report [29].

Differential gene expression analysis after 4 h of treatment revealed that approximately half of the transcripts significantly altered (41.3%) were shared between i-BET151 and i-BET858 (Fig. 2B, C and Additional file 2), indicating that whilst there may be a core gene network linked to BETi, there were significant differences between the effects of these two inhibitors. More interestingly, a high proportion of transcripts (53.4%) were found altered only when OVCAR-3 cells were treated with i-BET858, as opposed to a very low proportion (5.4%) that were uniquely affected with i-BET151 (Fig. 2C). Similar to the 4 h time-point effect, whilst 56.6% of the differentially regulated transcripts were common between the BETi treatments, again a relatively high proportion of genes (37.5%) were uniquely affected when cells were treated with i-BET858 for 24 h (Fig. 2B, C and Additional file 2). The disparity in transcript numbers may stem from the fact that i-BET858 is a more permeable BETi, and therefore, higher concentrations of i-BET151 should be able to mimic i-BET858’s transcriptomic signature.

Gene targets in the core network included well known BET targets such as NRG1, WNT5A, MAP3K3, CDKN1A/p21, MYCN, CDKN2B and the established BETi efficacy biomarker HEXIM1 [35, 36, 37] (Fig. 2E). To further evaluate the extent of the BET core network, we cross-referenced our results (4 h) with publicly available RNA-Seq datasets from OVCAR-3 cells treated with BETi JQ1 (0.125 µM, 40 min) [37]. Some of the BET targets (WNT5A, CDKN1A) were also identified in the JQ1 dataset, confirming the presence of a core gene set response to BETi in OC (Fig. 2D). Notably, the expression of the recognised BETi predictive marker c-MYC was unaltered after i-BET858 and i-BET151 treatment. These results were corroborated using qRT-PCR in the panel of HGSC cell lines (Fig. 2F).

Gene set enrichment (GSEA) pathway analyses of both i-BET151 and i-BET858 revealed prominent roles in the regulation of DNA damage response and canonical signalling pathways including mTORC1, TGFβ and IL6-JAK-STAT3 (Fig. 2G). Interestingly, i-BET151 appeared to down-regulate a gene set associated with the cellular response to doxorubicin after 4 h (Fig. 2G), suggesting that the molecular action of BETi may resemble that of doxorubicin, known for its ability to induce apoptotic cell death in OC cells [38]. Analysis further revealed that i-BET858 4 h treatment caused a significant down-regulation in the transcription of genes associated with G2/M checkpoint (Fig. 2G), highlighting a potential role for i-BET858 in cell cycle regulation.

Gene over-representation pathway analyses of the transcripts uniquely affected with i-BET858 after 4 and 24 h (Fig. 2C) revealed similar roles to those observed when analysing the entire BETi transcriptomic response, including DNA damage, G2/M checkpoint and apoptosis related pathways (Fig. 2H). Very interestingly, they also revealed a significant over-representation of genes with transcription starting site regions enriched for motifs matching SMAD4, PSMB5, E4F1 and DIDO1 transcription factors (Fig. 2H). Whilst all four transcripts were present in OVCAR-3 cells, only DIDO1 was found differentially expressed after 4 h of i-BET858 treatment (Fig. 2B and E). These results suggest that DIDO1, previously associated with cell cycle and apoptotic pathways [39], may have a differential role in the enhanced efficacy of i-BET858 over i-BET151.

As BET proteins function by modulating the interaction between diacetylated peptides present in histone tails via the tandem bromodomains to regulate gene transcription, we sought to investigate the association between individual BET proteins and gene expression changes caused by i-BET858 using chromatin immune-precipitation (ChIP). Initially, meta-analysis of publicly available ChIP-Seq datasets from OVCAR-3 cells treated with JQ1 (0.125 µM, 40 min, anti-BRD4) [37] were cross-referenced with our RNA-Seq results (i-BET8585, 1 µM, 4 h). Using this approach, we identified a set of genomic loci within the proximal promoter region of significantly down-regulated genes TOPBP1, ZHX2, BCL2L1 and CCR1, which were subsequently analysed using ChIP-qRT-PCR (Additional file 1: Fig. S1). i-BET858 treatment caused minor changes in BRD2, BRD3 and BRD4 enrichment in the studied promoter regions, suggesting that whilst i-BET858 was able to modulate gene expression, this was not due to displacement of BRD proteins at the loci investigated.

### i-BET858 enhances G2/M checkpoint arrest

**Fig. 3:**
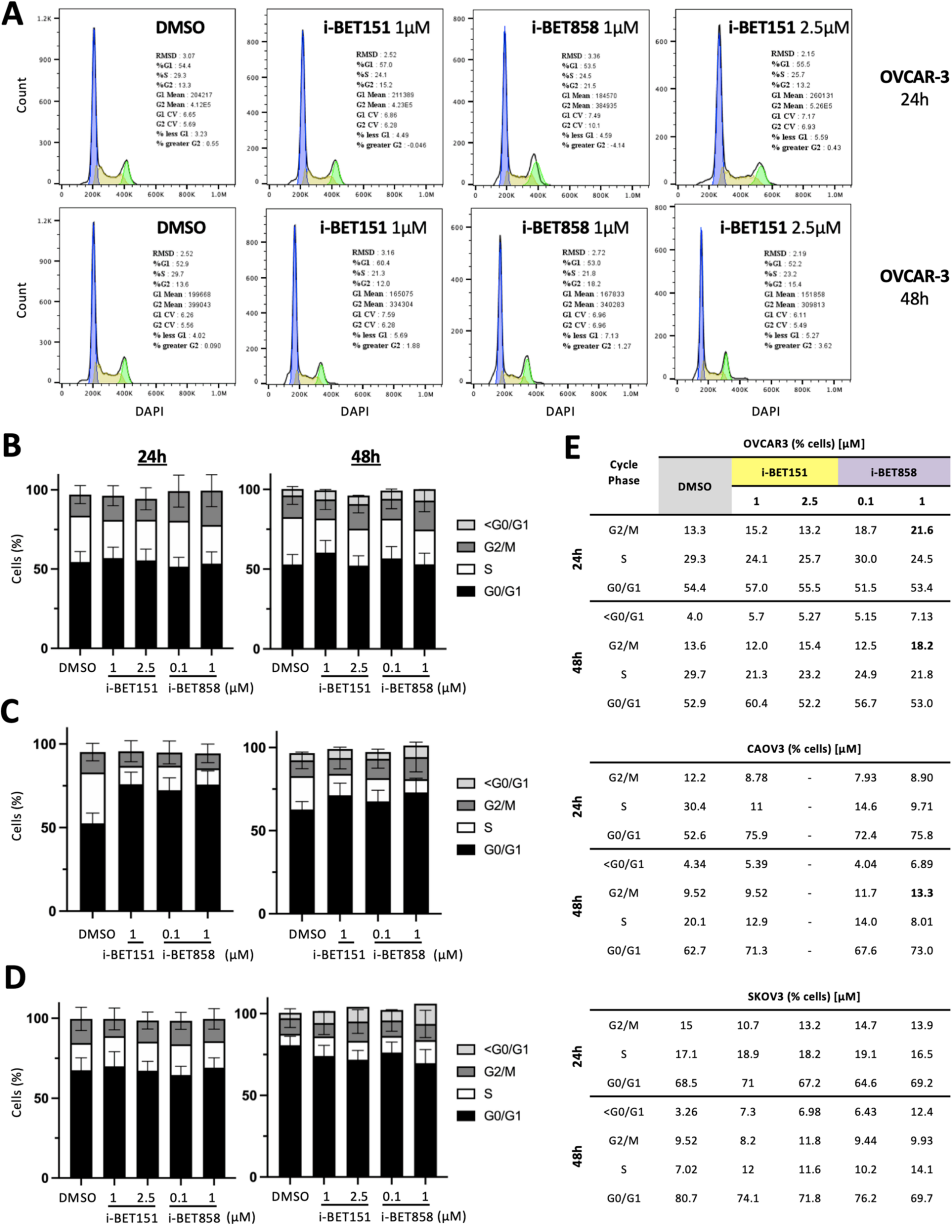
i-BET858 treatment leads to significant G2/M cell cycle arrest**. A** Flow cytometry cell cycle analyses of OVCAR-3 cells treated with different concentrations of i-BET151, i-BET858 and DMSO vehicle control for 24 and 48 h. Blue peaks represent cells in G0/G1 phase, whilst green peaks represent cells in G2/M phase. The area depicted as yellow represents cells in S phase. **B**–**D** Illustrative representations of the percentage of OVCAR-3, CAOV3 and SKOV3 cells present in different cell cycle phases following treatment. Particles containing less DNA than that of G0/G1 cells (< G0/G1) are related to apoptotic damage; due to low numbers this population was overlooked in graphs displaying 24 h treatment results. FlowJo™ was unable to fit peaks obtained at high concentrations of i-BET151 (2.5 µM) in CAOV3 cells; these datasets are included in Additional file 1: Fig. S2. **E** Table detailing specific percentages of cells detected per cell cycle phase following treatments

Based on the action of i-BET858 on genes whose products are involved in G2/M checkpoint (Fig. 2G), the effect on cell cycle was examined by flow cytometry (Fig. 3 and Additional file 1: Figs. S2 and S3). After 24 h, a significant increase in the number of cells at the G2/M phase was observed with 1 µM i-BET858 treatment (21.6%), whereas the accumulation of cells in G2/M was much lower even with an increased dose of 2.5 µM for i-BET151 (13.2%) (Fig. 3A, B and E). Similar differences were observed after 48 h: i-BET858 (1 µM, 18.2%), i-BET151 (2.5 µM, 15.4%) (Fig. 3A, B and E), suggesting that i-BET858 has a more prominent effect on causing G2/M cell cycle growth arrest. In CAOV3 cells, i-BET858 also led to a slight increase in G2/M populations after 48 h (1 µM, 13.3%), whilst i-BET151 had a less significant effect (9.52%) (Fig. 3C and E). Due to the presence of a sole peak, probably because of significant G0/G1 arrest, the cell cycle profile for i-BET151 (2.5 µM) did not fit the cell cycle model (Additional file 1: Fig. S2). Both BETi triggered a significant increase in the number of cells at G0/G1 after 24 h in CAOV3 cells (i-BET858, 100 nM, 75.8%; i-BET151, 1 µM, 75.9%) (Fig. 3C and E), consistent with previous reports [28, 29, 40]. A modest increase in the G0/G1 population was also observed in OVCAR-3 treated with 1 µM i-BET151 for 48 h, however no significant changes were detected in SKOV3 (Fig. 3 and Additional file 1: Fig. S3).

### i-BET858 induces γH2A.X and cleaved PARP

**Fig. 4:**
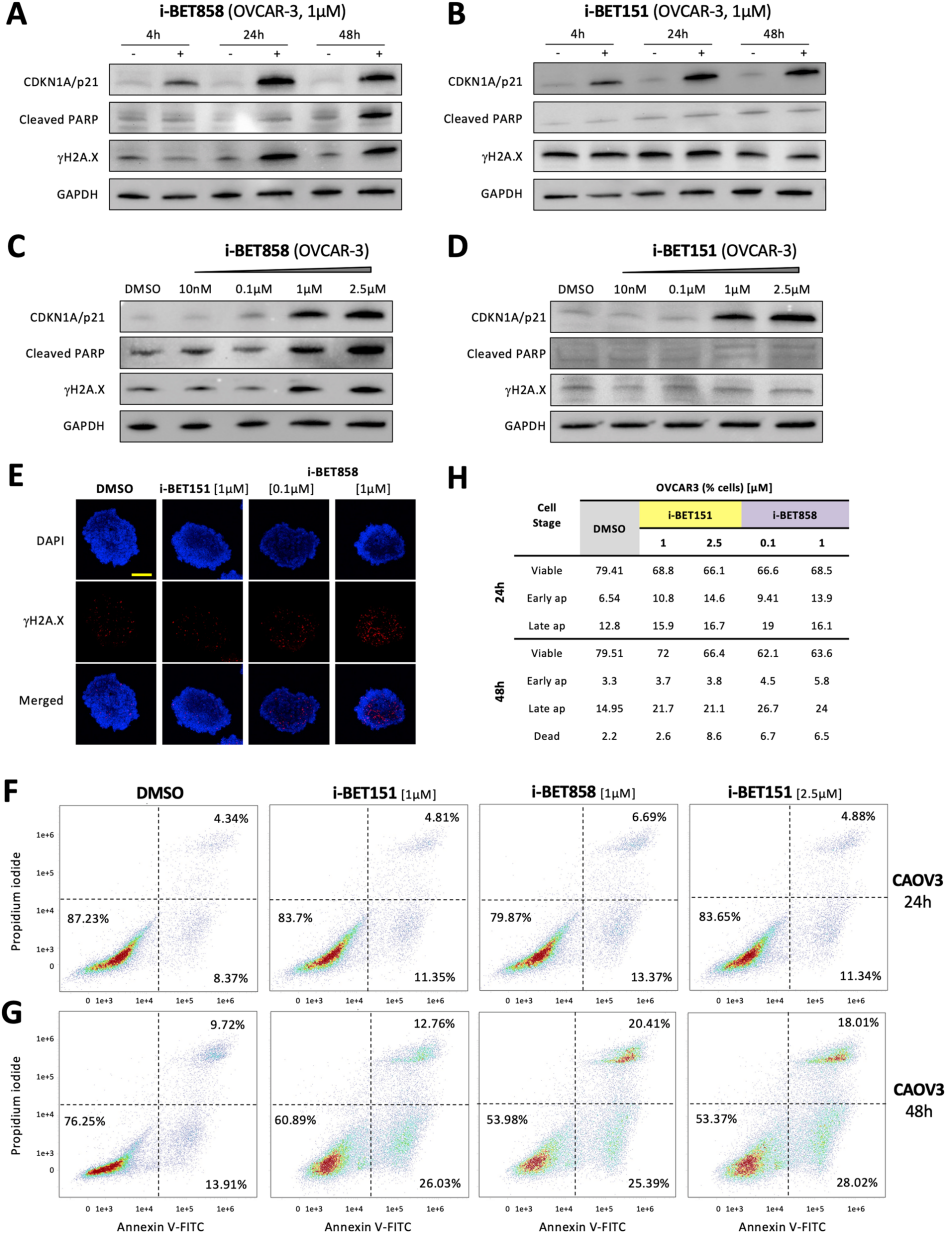
Ovarian cancer cells undergo apoptosis following i-BET858 treatment. Protein lysates of OVCAR-3 cells treated with (**A**) i-BET858 and **B** i-BET151 were subjected to western blot analyses to study changes in CDKN1A/p21, cleaved PARP and γH2A.X protein levels after 4, 24 and 48 h of treatment; GAPDH was used as loading control. **C**, **D** Protein lysates of OVCAR-3 cells treated with different concentrations of i-BET858 and i-BET151 (10 nM-2.5 µM; 48 h) were subjected to western blot analyses to study dose-dependent changes in CDKN1A/p21, cleaved PARP and γH2A.X protein levels. **E** Confocal microscopy images of OVCAR-3 spheroids treated with i-BET151, i-BET858 and DMSO control for 48 h. First and second rows show fluorescent-labelled DNA and γH2A.X staining, respectively; third row displays merged images of both fluorescent signals. Scale represents 100 µm. **F**, **G** Flow cytometry apoptosis analysis of CAOV3 cells treated with different concentrations of i-BET151, i-BET858 and DMSO vehicle control for (**F**) 24 h and **G** 48 h. Cells were stained with propidium iodide and Annexin V-FITC rendering 4 populations: viable (−, −), early apoptotic (−, +), late apoptotic (+, +) and dead (+, −), two of which are highlighted in the panels. Graphs display cell densities, whereby red, green and blue colours indicate high, medium and low cell densities, respectively. **H** Table detailing specific percentages of OVCAR-3 cells detected in each population following treatments. Percentages of dead cells after 24 h treatment were not significant and are not included in this table

Pathway analysis indicated a role in DNA damage for both BETi, which is consistent with previous reports demonstrating the down-regulation of DNA repair genes, and thus the attenuation of the response to DNA damage in BRCA wild-type OC [41, 42, 43] . To confirm the effects of BETi on the DNA damage repair machinery of OC cells, the presence of phosphorylated histone variant H2A.X (γH2A.X) was evaluated. Histone variant H2A.X constitutes 2–25% of the H2A histones in mammalian chromatin [44], and its rapid phosphorylation on Ser-139 occurs in response to the formation of DNA double-strand breaks [45]. In OVCAR-3 cells, γH2A.X levels increased in a time/dose-dependent manner following i-BET858 treatment, whereas no effect was observed with i-BET151 (Fig. 4A–D). These results suggest that i-BET858 achieved increased levels of DNA repair gene down-regulation, which led to the accumulation of DNA double-strand breaks marked by γH2A.X. This increase was also observed in 3D spheroid models by confocal microscopy (Fig. 4E), where the accumulation of γH2A.X foci following i-BET858 treatment surpassed that observed with i-BET151.

Our RNA-Seq analysis also identified a role for the BETi in apoptosis, which was validated using an Annexin 5-FITC and propidium iodide (An5/PI) staining assay. After 24 h of treatment both i-BET858 (1 µM) and i-BET151 (2.5 µM) induced a significant albeit only modest increase in early apoptosis in CAOV3 (i-BET858: 13.4%, i-BET151: 11.3%) and OVCAR-3 (i-BET858: 13.9%, i-BET151: 14.6%), and similarly after 48 h (Fig. 4F and H). This effect was more profound in SKOV3 cells after 24 h, where both i-BET151 (20.6%) and i-BET858 (30.5%) increased the percentage of early apoptotic events significantly in comparison with DMSO control (11.9%) (Additional file 1: Fig. S3). These results are in agreement with previous reports showing BETi can trigger apoptosis in OC [28, 29, 30, 31, 35]. As with the enhanced effect seen for G2/M cell cycle arrest, i-BET858 treatment resulted in a higher number of late apoptotic cells (1 µM; CAOV3: 20.4%; OVCAR-3: 24%; SKOV3: 13.4%) compared to i-BET151 even when the concentration of i-BET151 was increased: CAOV3 (1 µM: 12.8%; 2.5 µM: 18%), OVCAR-3 (1 µM: 21.7%; 2.5 µM: 21.1%) and SKOV3 (1 µM: 10.7%; 2.5 µM: 11.7%) (Fig. 4G, H and Additional file 1: Fig. S3).

Very importantly, when we analysed OVCAR-3 protein lysates to confirm the expression of known apoptotic markers including CDKN1A/p21 and cleaved PARP, we observed that whilst levels of CDKN1A/p21 protein were increased by both i-BET858 and i-BET151, cleaved PARP levels only increased following i-BET858 treatment (Fig. 4A–D). These results further support the notion that the cytotoxic activity of i-BET858 in OC cells is based on the activation of DNA damage-induced cell cycle arrest followed by apoptotic cell death. Both BETi induced PARP cleavage in CAOV3 and SKOV3 cells, yet increased doses of i-BET151 (2.5 µM) were necessary to attain cleavage levels comparable to those of i-BET858 (1 µM), again demonstrating the enhanced role of this agent (Additional file 1: Fig. S4). Interestingly, BRD proteins themselves were up-regulated following i-BET151 and i-BET858 treatments, suggesting a compensatory response to BETi (Additional file 1: Fig. S5) [46].

### BRD protein silencing only partially mimics the effect of BETi in OC cells

**Fig. 5:**
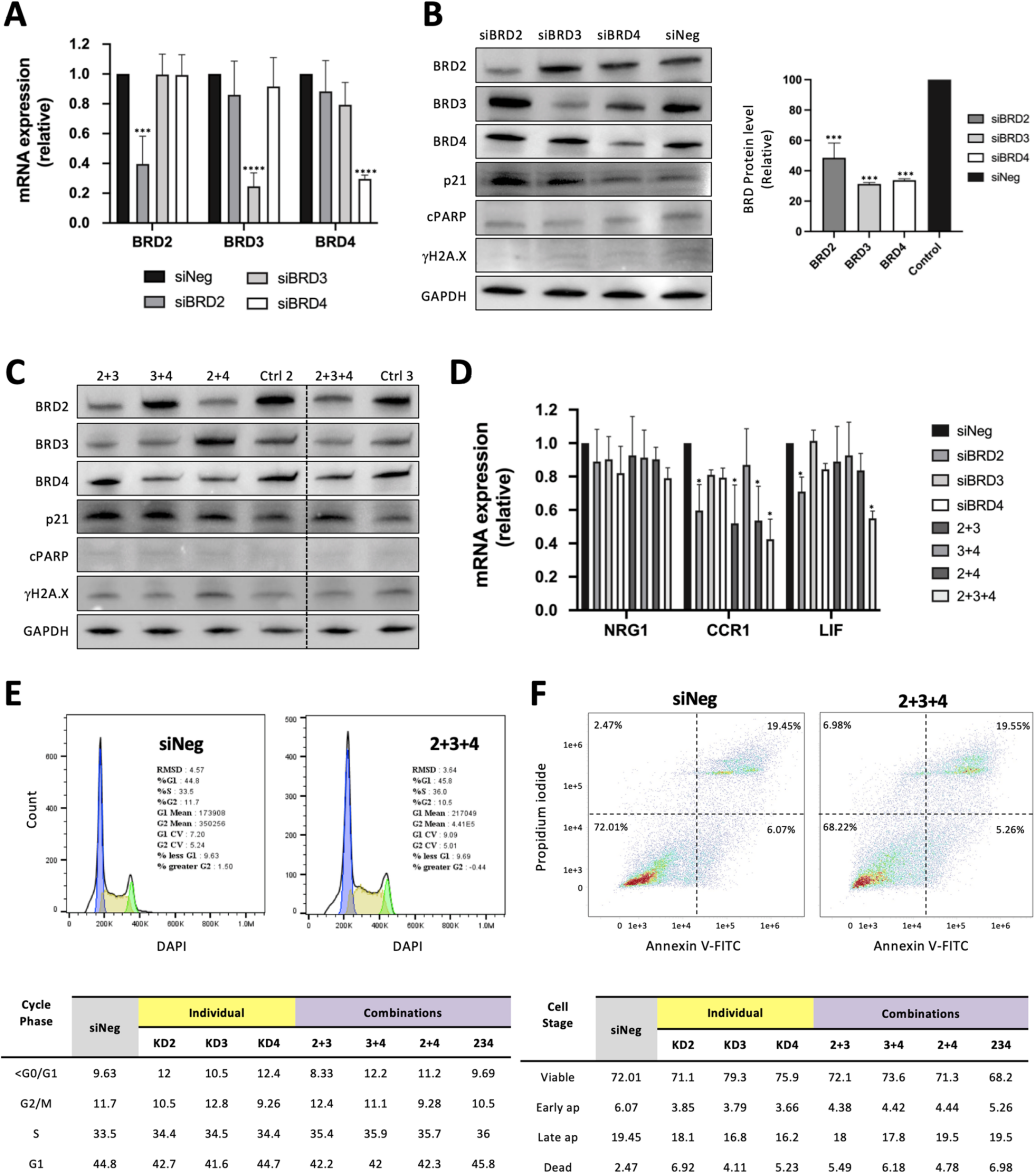
BRD protein knockdown effect on ovarian cancer BET-associated mechanisms. **A** siRNA-mediated knockdown in OVCAR-3 cells resulted in significant BRD2, BRD3 and BRD4 transcript down-regulation after 48 h compared to the control treatment (siNeg). **B** Protein lysates of OVCAR-3 cells treated with specific siRNAs were subjected to western blot analyses to study changes in BRD2, BRD3, BRD4, p21, cleaved PARP (cPARP) and γH2A.X protein levels after 48 h of knockdown (KD). The right panel displays proportional differences between relative densities of BRD proteins in KD and control samples calculated using ImageJ. **C** Protein lysates of OVCAR-3 cells treated with different combinations of siRNAs were subjected to western blot analyses. The two controls correspond to different amounts of scrambled siRNA introduced in cells to mimic the action of 2 or 3 target siRNAs (Ctrl 2 and Ctrl 3). **D** Cell lysates from OVCAR-3 cells treated with combinations of siRNAs (2, 3 and 4) for 48 h were subjected to qRT-PCR to study mRNA expression changes of i-BET858 targets NRG1, CCR1 and LIF. Each KD was compared to their correspondent control sample which included different amounts of scrambled siRNA; only one control was plotted to simplify (siNeg). **E** Flow cytometry apoptosis analysis of OVCAR-3 cells treated with siRNA targeting BRD2, BRD3 and BRD4 for 48 h. Cells were stained with propidium iodide and Annexin V-FITC rendering 4 populations: viable (−, −), early apoptotic (−, +), late apoptotic (+, +) and dead (+, −). Graphs display cell densities, whereby red, green and blue colours indicate high, medium and low cell densities, respectively. **F** Flow cytometry cell cycle analyses of OVCAR-3 cells treated with siRNA targeting BRD2, BRD3 and BRD4 for 48 h. Blue peaks represent cells in G0/G1 phase, whilst green peaks represent cells in G2/M phase. The area depicted as yellow represents cells in S phase. All values represent the mean ± SD of three biological samples (*P < 0.05, ***P < 0.001, ****P < 0.0001)

As described above, BETi have broad effects through targeting the BET proteins BRD2, BRD3 and BRD4 [19, 20, 21]. To determine whether i-BET858 functions through these canonical mechanisms, BRD2, BRD3 and BRD4 expression was suppressed in OVCAR-3, CAOV3 and SKOV3 cells using a small interfering RNA (siRNA) knockdown approach (KD). siRNA treatment resulted in significant decreases of BRD2, BRD3 and BRD4 after 48 h at both the mRNA and protein levels (Fig. 5A, B and Additional file 1: Fig. S6). Additionally, all possible siRNA KD BRD2, BRD3 and BRD4 permutations were also investigated since BETi effects associated with activation of apoptosis may be due to inhibitory action upon one, two or all three BET proteins (Fig. 5C and Additional file 1: Fig. S6).

BETi-associated targets previously identified via RNA-Seq were used to examine if BET KD mimicked the effects of i-BET858 and i-BET151 at the transcriptomic level (Fig. 5D and Additional file 1: Fig. S7). In OVCAR-3, CCR1 was down-regulated following BRD2 KD, either alone or in combination with BRD3 and BRD4 (Fig. 5D), suggesting a prominent role of BRD2 in modulating CCR1 expression. However, it was necessary to inhibit the expression of BRD2, BRD3 and BRD4 to achieve down-regulation of LIF (Fig. 5D). Whilst no changes in gene expression were observed in CAOV3, NRG1 was significantly down-regulated following individual BRD4 KD in SKOV3 cells (Additional file 1: Fig. S7).

Analysis of BRD2, BRD3 and BRD4 KD on cellular mechanisms associated with BETi in OC: cell cycle progression, apoptosis and DNA damage were undertaken to study the effect of BET silencing. Similar to the effect observed with i-BET858 and i-BET151, the individual KD of BRD2 and BRD3, as well as all KD combinations, resulted in the up-regulation of CDKN1A/p21 in OVCAR-3 (Fig. 5B and C) but not in SKOV3 (Additional file 1: Fig. S7). However, DAPI staining and flow cytometry analysis revealed that neither individual KD nor KD combinations were able to significantly increase the population of cells in G0/G1 phase (siBRD2: 42.7%; siBRD3: 41.6%; siBRD4: 44.7%; 2 + 3: 42.2%; 3 + 4: 42%; 2 + 4: 42.3%; 2 + 3 + 4: 45.8%; control: 42.9%) (Fig. 5E). The absence of any significant apoptotic events was further confirmed using An5/PI stain and flow cytometry analysis. The percentages of late apoptotic cell populations as a result of BET KD were similar to those of the control after 48 h (siBRD2: 18.1%; siBRD3: 16.8%; siBRD4: 16.2%; 2 + 3: 18%; 3 + 4: 17.8%; 2 + 4: 19.5%; 2 + 3 + 4: 19.5%; control: 19.45%) (Fig. 5F). Overall, these results suggest that the phenotypical effects of KD only partially mimic those observed following i-BET858 and i-BET151 treatment.

## Discussion

HGSC represents the most common and aggressive subtype of OC, and there is a clear need for improved therapeutic strategies. Several lines of evidence support the use of BETi, some of which have been evaluated in phase I/II clinical trials [22]. Here, we describe the cytotoxic profile and molecular mechanism of action i-BET858, a novel BETi that has not previously been evaluated in solid tumour models. The data presented herein suggest that i-BET858 has a significantly higher cytotoxic activity compared to other BETi and could be selected to pursue further in vivo investigations.

The main rationale for the use of BETi as therapeutic agents against OC is the observation of recurrent somatic, focal copy number amplifications of BRD4 in a significant percentage of HGSCs [47, 48]. Consistent with BRD4 amplification, increased levels of BRD4 mRNA have been observed in patient derived OC cells [49], however, no correlation has been found linking higher BET expression levels with increased BETi sensitivity [26]. Our studies conducted in patient derived clinical samples indicate that there is no significant correlation between BET expression levels and i-BET858 sensitivity, suggesting that the role of BET proteins in HGSC might be defined by alternative factors beyond BRD protein levels. Another common trait that provides further rationale for the use of BETi against HGSC is the recurrent amplification of c-MYC (~ 20–30%) [50], as c-MYC is a recognised predictive marker of response to BETi in haematological cancers [51]. In this study, i-BET858 treatment did not elicit consistent and significant c-MYC down-regulation, agreeing with previous data that pointed towards a haematological cancer-specific role of c-MYC as a marker of BETi [52].i-BET858 treatment resulted in a bipartite effect at transcriptomic level, where a core set of differentially expressed genes were shared with i-BET151, with an additional set of approximately 1300 genes regulated uniquely by i-BET858 in our ovarian cancer model. Specifically, the core set of genes included up-regulated HEXIM1, a well-established biomarker of BETi efficacy in OC [35], as well as kinases such as MAP3K3 and cell cycle regulators like CDKN1A/p21 and CDKN2B [30, 35]. Furthermore, both i-BET858 and i-BET151 induced the significant down-regulation of NRG1 [33], MYCN [53] and stem-related genes like WNT5A [37]. The activation or suppression of these targets had been previously linked with BET inhibition in OC, suggesting that at least part of the transcriptomic signature associated with i-BET858 is somewhat analogous to that of other BETi. However, significant differences were also noted, we did not observe down-regulation of ALDH1A1, previously linked to the action of BETi JQ1 and i-BET726 [37], SWI/SNF remodelling complexes [33] or the FOXM1 family (FOXM1, AURKA, PLK1) [29], highlighted as a major downstream signalling pathway of BETi in OC.

Gene over-representation analyses of the exclusive i-BET858 transcriptomic signature highlighted the enrichment of genes containing binding motifs for SMAD4, PSMB5, E4F1 and DIDO1. The SMAD4 protein is an integral part of the TGFβ pathway, known to act as a transcriptional repressor of p21 in ovarian carcinoma cells [54]. Whilst a synthetic lethality interaction between BETi (OTX-015, i-BET151, JQ1) and SMAD4 expression has been reported in colorectal cancer cells [55], their potential association in OC has not been investigated thus far. Similarly, the anti-proliferative effects of the member of the proteasome PSMB5 and the transcription factor E4F1 have been widely studied in triple negative breast cancer and myeloid leukaemia cells, respectively  [56, 57], whereas their potential links with BET inhibition as well as their wider roles in OC are largely unknown. Lastly, the death-inducer obliterator or DIDO1 is a putative transcription factor with known apoptotic functions, highly expressed in several diseases including bladder cancer [58] and oesophageal squamous cell carcinoma [59]. Whilst the regulation of DIDO1 via BET protein interactions had not been reported before, the significant down-regulation of this target following i-BET858 treatment, together with the significant over-representation of down-regulated DIDO1-target genes, strongly suggests that this putative transcription factor may have an important role in the unique response to i-BET858 in OC cells.

Preclinical data report that BETi attenuates OC cell growth via inducing G1 cell cycle arrest that forces cells to undergo senescence or apoptosis [30, 40, 60]. In agreement with previous studies, we observed apoptotic cell death as a result of i-BET858 treatment in OVCAR-3, CAOV3 and SKOV3 cells. However, whilst flow cytometry analysis following i-BET858 treatment in CAOV3 cells identified a significant increase in the population of G0/G1 cells, which usually implies G1 cell cycle arrest [61], such phenomenon was not observed in OVCAR-3 or SKOV3 cells. In contrast, the effect of i-BET858 on cell cycle progression in OVCAR-3 cells was associated with the induction of G2/M cell cycle arrest, an event that has been linked with BETi in previous studies but never in OC cells [22, 62].

In addition to cell cycle and apoptosis, BETi such as JQ1 or lNCB054329 have been reported to induce DNA damage in OC cell lines, including SKOV3 and OVCAR-3 [41, 42, 43]. This effect is a consequence of BETi-associated DNA repair gene down-regulation and is characterised by the accumulation of histone γH2A.X [45]. In line with this, we observed the significant activation of histone variant γH2A.X as a result of i-BET858 treatment in CAOV3, OVCAR-3 and SKOV3 cells. In our study, the presence of γH2A.X was consistently detected at an earlier stage (24 h) compared with apoptosis marker cleaved PARP (48 h), suggesting a drug mechanism of action that initiates with the accumulation of DNA damage as a consequence of the down-regulation of DNA repair-related genes. Based on our findings, the accumulation of DNA damage would prompt G1 cell cycle arrest, eventually leading to apoptotic cell death. However in OVCAR-3 cells, known to carry a cyclin E1/CCNE1 amplification [63], it is possible that higher basal levels of cyclin E1 may account for their ability to avoid G1 cell cycle arrest. In this case, cells with increased DNA damage would move on to the G2 phase of the cell cycle, only to accumulate upon G2 checkpoint and eventually ending in apoptotic cell death.

Our observations indicate that single and/or multiple BET protein KD may not mirror the effect of BETi because such compounds interfere with binding of BET proteins to chromatin but do not reduce BET protein expression. In fact, we and others have shown that BETi increases BET protein expression [46], suggesting an intrinsic compensatory effect that eventually leads to the observed phenotypical drug response with respect to cell cycle, apoptosis and DNA damage.

Our studies have shown that i-BET858 has strong cytotoxicity profiles against OC cell lines regardless of BRCA mutational status, consistent with previous reports on BETi like lNCB054329 or JQ1 [41, 42, 43] . These results suggest that i-BET858 is able to impair homologous recombination capacity and thus the response to DNA damage in both BRCA wild-type and mutated ovarian cancer cells. It will be interesting to test whether i-BET858 exhibits a synergistic increase in DNA damage in BRCA wild-type cells combined with PARP inhibitors. Whilst in vitro nanomolar IC_50_ values displayed in HGSC cell lines were not observed in primary cells, i-BET858 cytotoxicity profiles were consistently lower than those obtained with i-BET151 or i-BET726. These three compounds have shown similar intrinsic cellular potencies [21, 34], suggesting that our results may stem from an enhanced cellular permeability of the drug, an observation supported by the higher efficacy of i-BET858 at disrupting cell line spheroids.

## Conclusions

Our work on i-BET858 exposed several phenotypical enhancements, including increased DNA damage, cell cycle arrest and apoptosis, which suggest that i-BET858 may represent an alternative option to target BET proteins in HGSC.

## Methods

### Cell culture

Ovarian cancer cell lines were purchased from ATCC® (LGC Ltd). SKOV3 (CVCL_0532) and OVCAR-3 (CVCL_0465) were maintained in RPMI 1640 media (Gibco™, 11875093) supplemented with 20% foetal calf serum (FBS; Gibco™, 10270106) and 10 μg/ml of insulin solution from bovine pancreas (Sigma, I0516). UWB1.289 (BRCA1mut; CVCL_B079) and CAOV3 (CVCL_0201) were maintained in DMEM/F-12 + GlutaMAX™ (Thermo Scientific, 31331093) (10% FBS). None of the cell lines we have used are frequently misidentified. Patient derived primary ovarian cancer cells were isolated from ovarian biopsies using a protocol adapted from Shepherd et al. [64]; the main difference is the use of Collagenase type I (2 mg/mL; Sigma, 17100–017) instead of Dispase II. Primary cells were maintained in 50% MCDB 105 (Sigma, M6395) + 50% M199 (Gibco, 31150–022) (20% FBS). All media was supplemented with penicillin [100 U/ml] and streptomycin [100 μg/ml] (P/S; Gibco, 15140–122), and cells were maintained at 37 °C in a humidified 5% CO_2_ incubator. 3D spheroids were grown in 96-well ultra-low attachment (ULA) plates (Corning, CLS4520). The human biological samples were sourced ethically, and their research use was in accord with the terms of the informed consents under an IRB/EC approved protocol. Mycoplasma contamination was routinely tested with the MycoAlert Mycoplasma Detection kit (Lonza, LZLT07218).

### Antibodies

The following commercial antibodies were used for immune blotting with the indicated dilutions: anti-BRD2 (Cell Signalling, 5848, AB_10835146), anti-BRD3 (Bethyl, A302-368, AB_1907251), anti-BRD4 (Bethyl, A301-985, AB_2620184), anti-GAPDH (Santa Cruz, sc-47724, AB_627678), anti-CDKN1A/p21 (Cell Signalling, 2947, AB_823586), anti-cleaved PARP (Cell Signalling, 9541, AB_331426) and anti-γH2A.X (Cell Signalling, 2577, AB_2118010). Anti-γH2A.X (1:50, 2577) and Alexa Fluor 594 anti-rabbit secondary antibody (1:100, Thermo Scientific, A-11012) were used for spheroid confocal microscopy. Anti-BRD2 (5848), anti-BRD3 (A302-368) and anti-BRD4 (A301-985) were used for chromatin immuno-precipitation (ChIP).

### Cell viability assays

The RealTime-Glo™ MT Cell Viability Assay (Promega, G9712) was used to assess live cell viability of cells grown in 2D monolayers following manufacturer’s instructions. The CellTiter-Glo™ 3D Cell Viability Assay (Promega, G9682) was used to assess end-point cell viability of cells grown as 3D spheroids. All samples were tested in triplicates. The substrates were always added to un-treated wells and empty wells as additional controls. IC_50_ values were calculated using GraphPad Prism (V9). Raw luminescence values were transformed into logarithmic values, normalised and fitted to a dose–response curve using a non-linear regression; 100% and 0% values were defined by DMSO vehicle control (Sigma, D8418) and Staurosporine positive control (Tocris, 1285), respectively.

### RNA extraction, qRT-PCR and PCR arrays

Total RNA was isolated using the RNeasy Plus Mini Kit (Qiagen, 74136) and reverse transcribed using the high-capacity cDNA reverse transcription kit (Thermo Scientific, 4368814). All qRT-PCR reactions were conducted in a CFX96™ real-time PCR detection system (Bio-Rad) using iTaq™ Universal SYBR® Green supermix (Bio-Rad, 1725125). All samples were tested in triplicates. When possible, synthetic oligonucleotides (sequences available upon request) span exon-exon boundaries to preclude amplification of genomic DNA. Relative gene expression was determined following the ΔC_t_ method [65] and normalised to an internal reference gene (RPL5). One-way ANOVA statistical analyses were performed on ΔC_t_ values of three biological replicates using GraphPad Prism (V9); Sidak’s test was used to correct for multiple comparisons.

### RNA-Sequencing and in silico pathway analysis

Total RNA samples were sent to Novogene for library preparation (Eukaryotic mRNA library preparation – poly-A enrichment) and sequencing (NovaSeq PE150). Raw fastq files were quality-checked using FastQC [66], a quality-control tool for high throughput sequencing data. Reads were aligned to the GRCh38/hg38 genome build using STAR [67]; gene count tables were generated using the -quantMode GeneCounts argument in STAR. The DESeq2 median of means method was used to normalise the gene count tables to account for sample depth [68]. DESeq2 was used to correct for multiple hypothesis testing and determine significantly modified transcripts between control and experimental samples (FDR < 0.05). Raw and processed data are deposited in the GEO Database with accession number GSE174670. RNA-Seq data used for comparison was downloaded from the GEO Database (GSE77568) and analysed using STAR and DESeq2. The platform WebGestalt was used to perform gene set enrichment analyses and gene set over-representation analyses comparing lists of differentially expressed genes in each experimental condition against all major annotation datasets. The lists of genes were compared using Venn diagrams through the Venny website [69]. Principal component analysis (PCA) were generated using the package factoextra [70]. Volcano plots and pathway analysis graphs were generated using the data visualisation package gglot2 [71].

### Protein extraction and western blot

Cells were lysed using RIPA buffer (Sigma, R-0278) including 1X Halt™ Protease and Phosphatase Inhibitor Cocktail (Thermo Scientific, 1861281). Total protein was quantified using a standardised bovine serum albumin (BSA; PAN™-Biotech, P06-1391050) concentration curve following the Bio-Rad DC™ protein assay (Bio-Rad, 5000112). Total protein samples were separated using SDS-PAGE gels (Bio-Rad, 4568094) and then transferred to PVDF membranes (Bio-Rad, 1704156). Primary antibody incubations were performed overnight (O/N) followed by two hours in the presence of HRP-conjugated secondary antibodies (Cytiva; anti-mouse: NA931V, anti-rabbit: NA934V). Protein intensity was detected with Clarity™ Western ECL substrate (Bio-Rad, 170–5060). Relative protein intensity levels were calculated using ImageJ [72]. Graphical depictions of relative protein levels represent the proportional difference between a treatment and its control (100%). One-way ANOVA analyses were performed on relative intensity values using GraphPad Prism (V9); Sidak’s test was used to correct for multiple comparisons.

### Flow cytometry

All flow cytometry experiments were undertaken using a CellStream® (Luminex). Cell cycle staining was performed using DAPI ready-made solution (1 µl/mL; Sigma, MBD0015) following manufacturer’s instructions. Prior to staining, cells were fixed in 70% ethanol (-20 °C, 1 h). Cell cycle profiles were analysed using FlowJo™ 10 using the Watson (Pragmatic) model; schematic representations of cell percentages in different cell cycle phases were graphed using GraphPad Prism (V9). Apoptosis staining was performed using Annexin V-FITC (BioLegend, 640906) and propidium iodide (PI, BioLegend, 421301) as per manufacturer’s instructions, which included the use of Annexin V Binding Buffer (BioLegend, 422201) and Cell Staining Buffer (BioLegend, 420201). Unstained cells and cells stained with Annexin V/PI only were used to calculate the compensation matrix that was applied to all the data to adjust for signal overlap between channels of the emission spectra.

### RNA interference and ChIP

ON-TARGETplus BRD2/BRD3/BRD4 siRNA SMARTpools (Horizon Discovery: L-004935–00-0005, L-004936–00-0005, L-004937–00-0005) were used in order to transiently knockdown BRD2, BRD3 and BRD4, whilst a ON-TARGETplus non-targeting pool (Horizon Discovery, D-001810–10-05) was used as control. (siRNA sequences can be found in Additional file 1: Table S1.) Target cells were transfected with siRNA pools [25 nM] using DharmaFECT 1 Transfection Reagent (Horizon Discovery, T-2001–01) following manufacturer’s instructions. ChIP-Seq data used for comparison were downloaded from the GEO Database (GSE77568) and analysed using Bowtie2 [73] and MACS2 [74]. Chromatin immuno-precipitation qRT-PCR (ChIP-qRT-PCR) experiments were performed using a Chromatrap® Pro-A kit as per manufacturer’s instructions (Porvair Plc, 500189), using 10 µg of chromatin and 5 µg of antibody per sample. Chromatin-antibody binding reaction was carried out at 4 °C (1 h).

### Confocal microscopy

Spheroid fixing and staining protocols were adapted from Weiswald et al., 2010 [75]. 40 spheroids were fixed and permeabilised in phosphate-buffered saline (PBS, Gibco) containing 4% paraformaldehyde (PFA; Chemcruz®, sc-281692) and 1% Triton X-100 (Sigma, 1001124827) for 3 h (4 °C). Spheroids were washed in PBS (3 times, 10 min; Gibco, 10010–015) and then de-hydrated in an ascending series of methanol concentrations diluted in PBS (25%, 50%, 75%, 95%) for 30 min each, followed by 2 h in 100% methanol (4 °C). Spheroids were then re-hydrated in the same descending series and washed in PBS (3 times, 10 min). In preparation for antibody staining, spheroids were blocked using PBS-T (PBS with 0.1% Triton X-100) containing 3% BSA O/N (4 °C). Spheroids were then incubated with primary antibody diluted in PBS-T for 72 h (4 °C), followed by secondary antibody incubation for 24 h (4 °C). Cell nuclei were eventually counter-stained with Hoechst in PBS (1:2000; Thermo Scientific, 33342) for 45 min at room temperature. Finally, spheroids in PBS were placed into 8-well chambers designed for immuno-fluorescence and high-end microscopy (Ibidi). Z-stack images were taken on a Zeiss LSM710 confocal microscope with a 10 × objective.

### Clinical samples

Ethical approval for processing ovarian patient samples has been obtained through Local Research Ethics Committee LREC Wales (ref 15/WA/0065) for the collection of biopsies from consented OC patients. Formal written consent was obtained from all patients at the time of recruitment into the study.

## Supplementary Information

**Additional file 1**: **Supplementary Figures and Tables**. Word document including Supplementary Figures S1-S7 and Supplementary Tables S1, S2.

**Additional file 2**: **Supplementary Table S3.** Excel file including lists of significantly up/down-regulated genes following BETi treatment and subsequent RNA-Sequencing.

## Data Availability

The datasets supporting the conclusions of this article are available in the GEO repository (GSE174670; https://www.ncbi.nlm.nih.gov/geo/query/acc.cgi?acc=GSE174670).
