# Supplementary material for: Bromodomain inhibitor i-BET858 triggers a unique transcriptional response coupled to enhanced DNA damage, cell cycle arrest and apoptosis in high-grade ovarian carcinoma cells
Source: Clin Epigenetics. 2023 Apr 15;15:63. doi: 10.1186/s13148-023-01477-x (PMC10105475; doi:10.1186/s13148-023-01477-x)
Supplement: Supplementary file 1 — Additional file 1: Supplementary Figures and Tables. Word document including Supplementary Figures S1-S7 and Supplementary Tables S1, S2. [file 13148_2023_1477_MOESM1_ESM.docx]

**Additional file 1**

**Supplementary Information**

**Supplementary Figures**

**Supplementary Figure S1. ChIP-qRT-PCR enrichment after i-BET858 treatment**


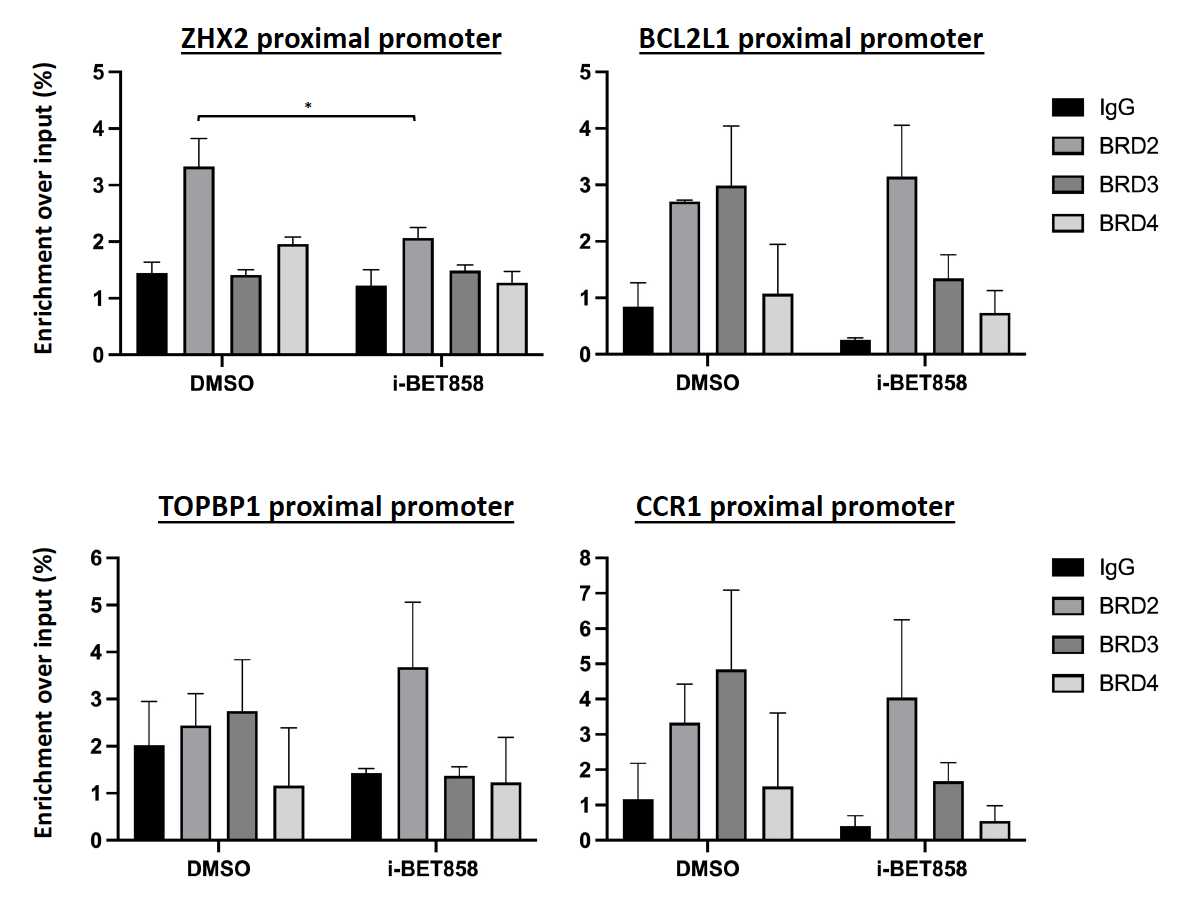


BRD2, BRD3 and BRD4 relative enrichment within proximal promoter regions of genes *ZHX2*, *BCL2L1*, *TOPBP1* and *CCR1* following i-BET858 treatment was analysed using ChIP-qRT-PCR. Prior to antibody enrichment, OVCAR-3 cells were treated with i-BET858 (1µM, 40 minutes) or DMSO; IgG was used as ChIP-qRT-PCR negative control. All values represent the mean ± SD of three biological samples (^*^p<0.05).

**Supplementary Figure S2. DNA content profiles following BETi treatment in CAOV3 and OVCAR-3 cells**

**
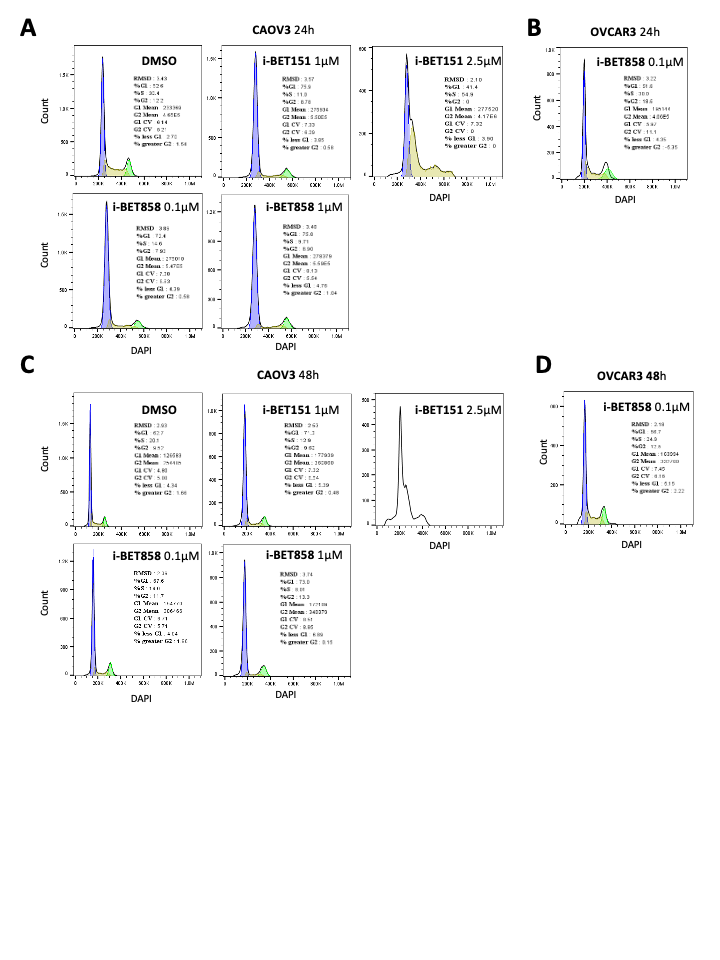
**

Flow cytometry cell cycle analyses of CAOV3 and OVCAR-3 cells treated with different concentrations of i-BET151, i-BET858 and DMSO vehicle control for 24h (A, B) and 48h (C, D). Blue peaks represent cells in G0/G1 phase, while green peaks represent cells in G2/M phase. The area depicted as yellow represents cells in S phase.

**Supplementary Figure S3. Flow cytometry analysis of DNA profile and apoptosis in SKOV3 cells following BET treatment**

**
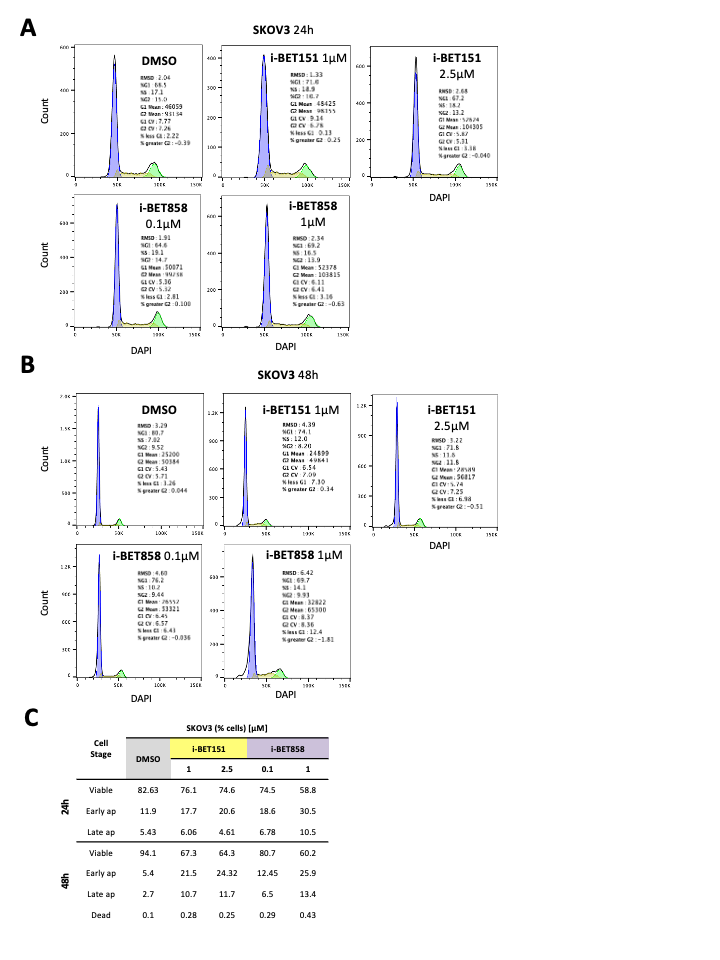
**

Flow cytometry cell cycle analyses of SKOV3 cells treated with different concentrations of i-BET151, i-BET858 and DMSO vehicle control for 24h (A) and 48h (B). Blue peaks represent cells in G0/G1 phase, while green peaks represent cells in G2/M phase. The area depicted as yellow represents cells in S phase. (C) Table detailing specific percentages of SKOV3 cells detected in each population of cells stained with Propidium iodide and Annexin V-FITC.

**Supplementary Figure S4. BETi triggers the activation of DNA damage and apoptotic markers in CAOV3 and SKOV3 cells**

**
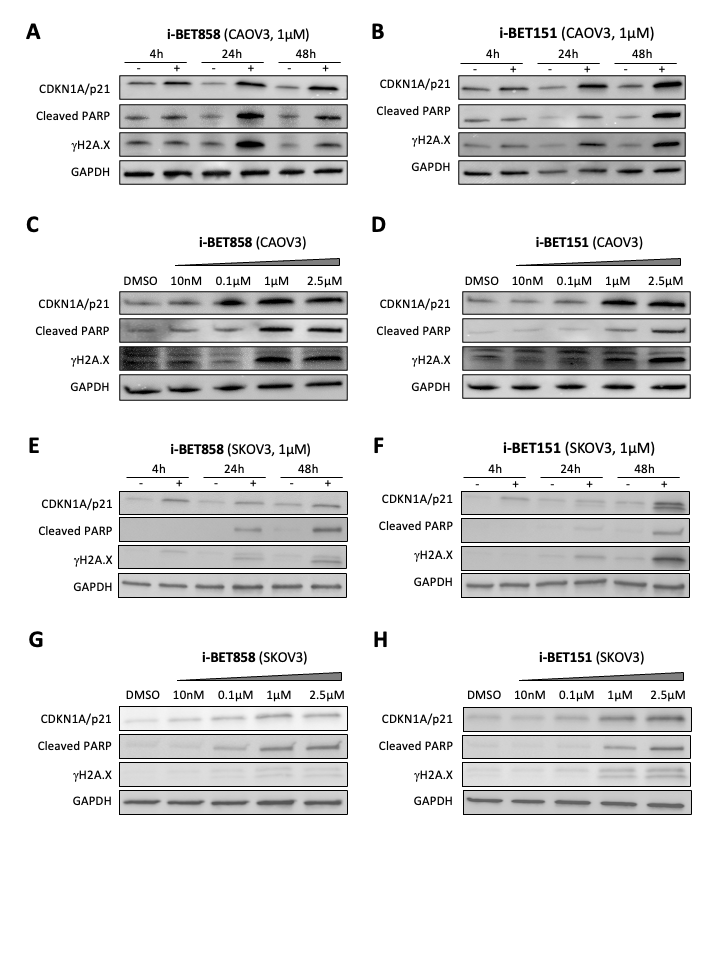
**

Protein lysates of CAOV3 (A, B) and SKOV3 (E, F) cells treated with i-BET858 (1µM), i-BET151 (1µM) and DMSO control (-) were subjected to western blot analyses to study changes in p21, cleaved PARP and γH2A.X protein levels after 4, 24 and 48 hours of treatment; GAPDH was used as loading control. Protein lysates of CAOV3 (C, D) and SKOV3 (G, H) cells treated with different concentrations of i-BET858 and i-BET151 (10nM-2.5µM) were subjected to western blot analyses to study dose-dependent changes in p21, cleaved PARP and γH2A.X protein levels.

**Supplementary Figure S5. BETi alters BRD protein expression levels in CAOV3 and OVCAR-3 cells**

**
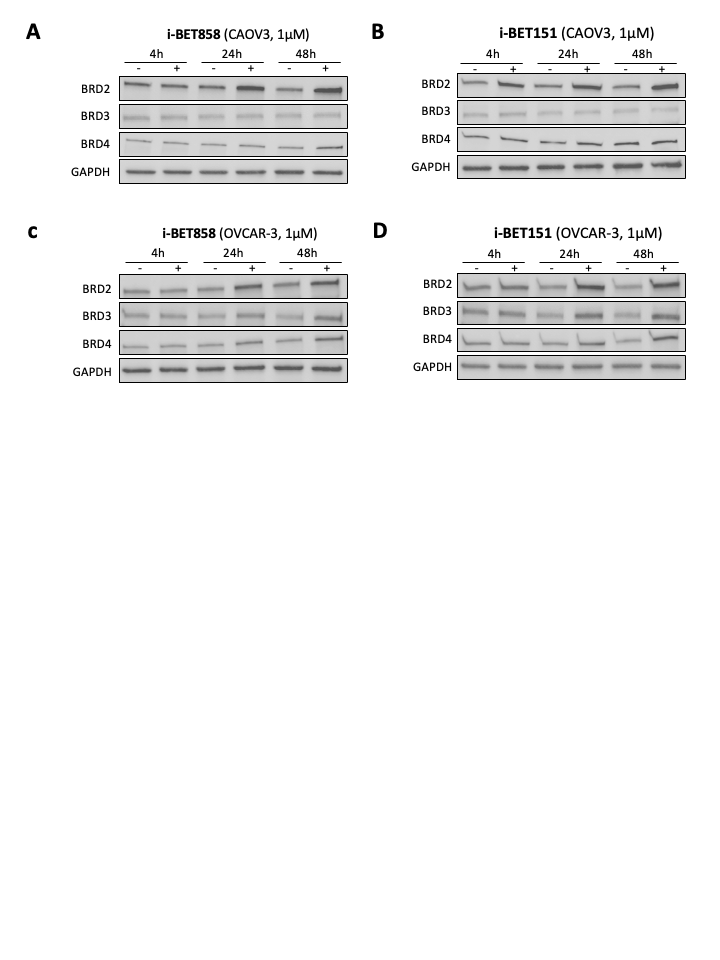
**

Protein lysates of CAOV3 (A, B) and OVCAR-3 (C, D) cells treated with i-BET858 (1µM), i-BET151 (1µM) and DMSO control (-) were subjected to western blot analyses to study changes in BRD2, BRD3 and BRD4 protein levels after 4, 24 and 48 hours of treatment; GAPDH was used as loading control.

**Supplementary Figure S6. Confirmation of BET protein knockdown in OVCAR-3, CAOV3 and SKOV3 cells**

**
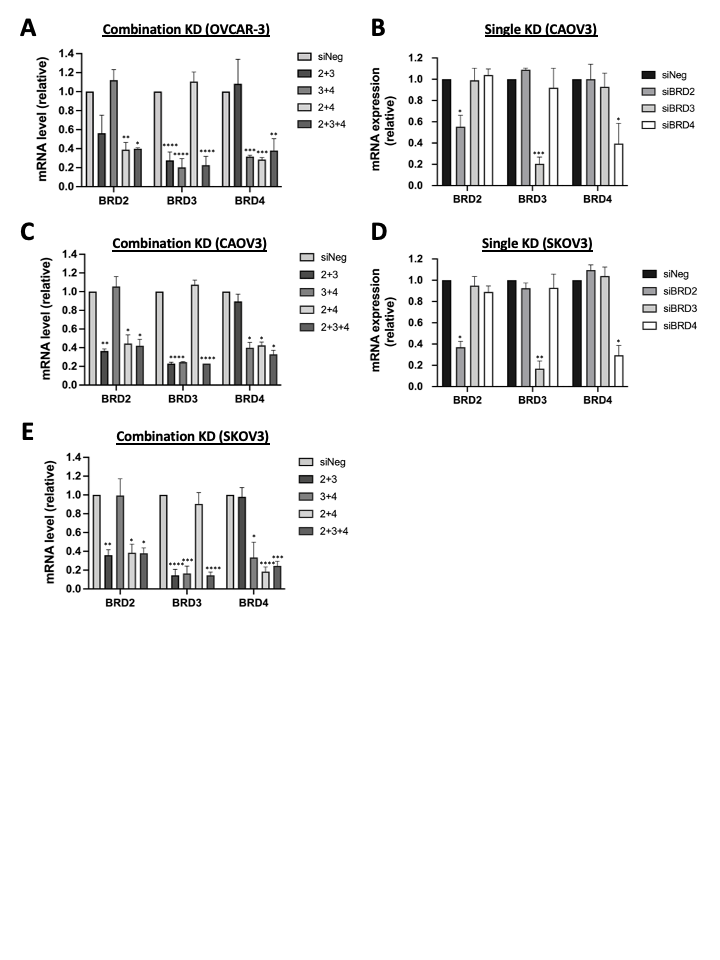
**

siRNA-mediated single and combinatorial knockdown (KD) in OVCAR-3 (A), CAOV3 (B, C) and SKOV3 (D, E) resulted in significant BRD2, BRD3 and BRD4 transcript down-regulation after 48h compared to the control treatments. Each KD was compared to their correspondent control sample which included different amounts of scrambled siRNA; only one control was plotted to simplify the images. All values represent the mean ± SD of three biological samples (^*^p<0.05, ^**^p<0.01, ^***^p<0.001, ^****^p<0.0001).

**Supplementary Figure S7. Effect of BET protein knockdown in studied cell lines**


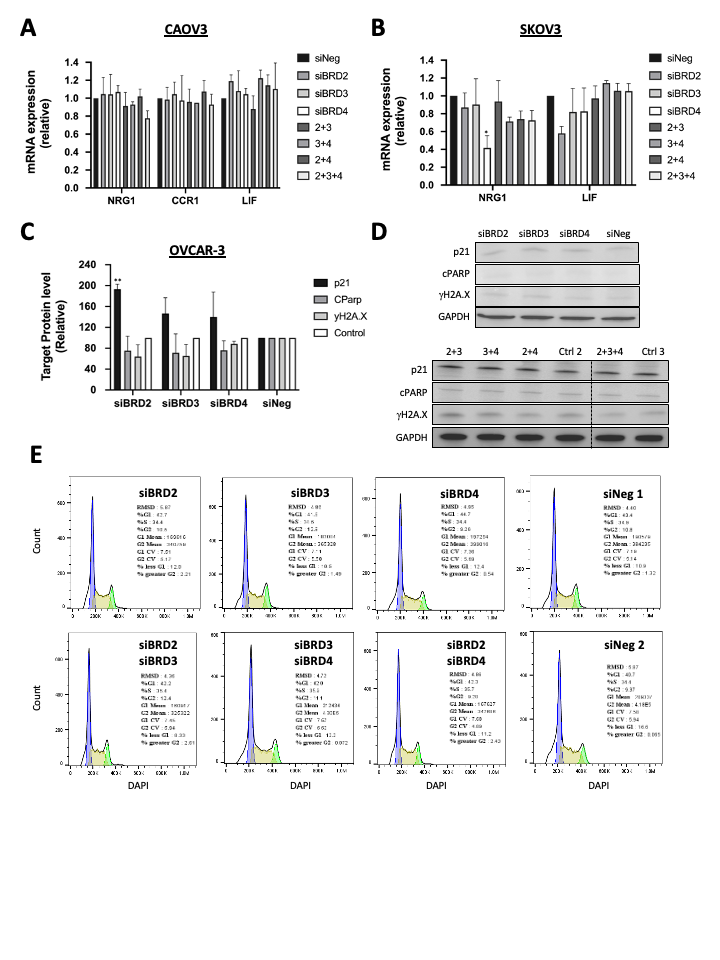


Cell lysates from CAOV3 (A) and SKOV3 (B) treated with single and combinatorial siRNAs for 48h were subjected to qRT-PCR to study changes of targets NRG1, CCR1 (except for SKOV3 as this target is not present) and LIF. Each KD was compared to their correspondent control sample; only one control was plotted to simplify (siNeg). (C) Proportional differences between relative densities of p21, cleaved PARP (cPARP) and γH2A.X in KD and control OVCAR-3 samples calculated using ImageJ. (D) Protein lysates of SKOV3 cells treated with single and combinatorial siRNAs were subjected to western blot analyses to study changes in p21, cPARP and γH2A.X levels after 48h (E) Flow cytometry cell cycle analyses of SKOV3 cells treated with siRNAs. All values represent the mean ± SD of three biological samples (^*^p<0.05, ^**^p<0.01).

**Supplementary Tables**

**Supplementary Table 1. List of the siRNAs used to target BRD2, BRD3 and BRD4**

| **Product** | **Target Sequence** | **Description** |
| --- | --- | --- |
| BRD2  ON-TARGETplus SMARTpool | 5’-CACGAAAGCUACAGGAUGU-3’ | siBRD2 |
|  | 5’-GGGCCGAGUUGUGCAUAUA-3’ |  |
|  | 5’-CCUAAGAAGUCCAAGAAAG-3’ |  |
|  | 5’-GUCCUUUCCUGCCUACGUA-3’ |  |
| BRD3  ON-TARGETplus SMARTpool | 5’-AAUUGAACCUGCCGGAUUA-3’ | siBRD3 |
|  | 5’-CGGCUGAUGUUCUCGAAUU-3’ |  |
|  | 5’-GGAGAGAUAUGUCAAGUCU-3’ |  |
|  | 5’-GCGAAUGUAUGCAGGACUU-3’ |  |
| BRD4  ON-TARGETplus  SMARTpool | 5’-AAACCGAGAUCAUGAUAGU-3’ | siBRD4 |
|  | 5’-CUACACGACUACUGUGACA-3’ |  |
|  | 5’-AAACACAACUCAAGCAUCG-3’ |  |
|  | 5’-CAGCGAAGACUCCGAAACA-3’ |  |
| ON-TARGETplus Non-targeting Pool | 5’-UGGUUUACAUGUCGACUAA-3’  5’-UGGUUUACAUGUUGUGUGA-3’  5’-UGGUUUACAUGUUUUCUGA-3’  5’-UGGUUUACAUGUUUUCCUA-3’ | Non specific siRNA |

**Supplementary Table 2. Primary cells obtained from patient derived clinical samples**

| **Sample** | **Age** | **Diagnosis** | **Stage** | **BRCA status** | **Chemotherapy** |
| --- | --- | --- | --- | --- | --- |
| HG1 | 68 | HGSC | IV | Unknown | 6 cycles |
| HG2 | 65 | HGSC | IVa | Unknown | 4 cycles |
| HG3 | 69 | HGSC | III | Unknown | 2 cycles |
| HG4 | 69 | HGSC | IIIc | Unknown | 6 cycles |
| HG5 | 59 | HGSC | IIIc | Unknown | Unknown |
| HG6 | 67 | HGSC | IIIc | Unknown | 6 cycles |
